# Design and fabrication of a stereo-video camera equipped unoccupied aerial vehicle for measuring sea turtles, sharks, and other marine fauna

**DOI:** 10.1371/journal.pone.0276382

**Published:** 2022-10-18

**Authors:** Susan E. H. Piacenza, Joseph R. Piacenza, Kenneth J. Faller, Nathan J. Robinson, Tabitha R. Siegfried

**Affiliations:** 1 Department of Biology, University of West Florida, Pensacola, FL, United States of America; 2 Department of Fisheries, Oregon State University, Wildlife and Conservation Sciences, Corvallis, OR, United States of America; 3 Department of Mechanical Engineering, University of West Florida, Pensacola, FL, United States of America; 4 College of Engineering, Oregon State University, Corvallis, OR, United States of America; 5 Department of Electrical and Computer Engineering, California State University, Fullerton, Fullerton, CA, United States of America; 6 Institut de Ciències del Mar, Spanish National Research Council (CSIC), Barcelona, Spain; 7 Cape Eleuthera Institute, Cape Eleuthera Island School, Eleuthera, The Bahamas; 8 Gulfarium C.A.R.E. Center, Gulfarium Marine Adventure Park, Fort Walton Beach, FL, United States of America; Institut de Recherche pour le Developpement, FRANCE

## Abstract

The recent commercialization of unoccupied aerial vehicles (UAVs) has facilitated their incorporation into a variety of ecological studies. While UAVs are able to provide accurate visual data of marine species from an aerial perspective, these devices have some limitations that make measuring marine animals below the surface challenging. Many marine organisms are often visible from the air, but are deeper in the water column, and current methods cannot measure animals below the surface. Here, we developed and tested a stereo-video camera (SVC) system that was mounted onto a commercially-available UAV. We used the SVC-UAV to conduct remote body-size measurements for two marine species: the green sea turtle (*Chelonia mydas*) and the nurse shark (*Ginglymostoma cirratum*). When comparing SVC measurements to those taken by hand, the SVC-UAV had a mean absolute error (MAE) of 4.44 cm (n = 6; mean percent error (MPE) = 10.6%) for green sea turtles and 7.16 cm absolute error (n = 1; PE = 3.6%) for the nurse shark. Using a linear model, we estimated the slope of the SVC versus hand measurements for green sea turtles to be 1.085 (±0.099 SE), and accounting for the standard error, a measurement bias was not apparent. Using model selection, based on a global model predicting MAE from animal distance to the SVC and body size, the top ranked model was the intercept-only model. This indicates that neither animal distance nor body size strongly influenced measurement error. Incorporating SVC systems into UAVs can allow for relatively accurate measurements of near surface-dwelling marine species. To our knowledge, there is no other stand-alone SVC for UAVs available that offers similar accuracy and utility.

## Introduction

Unoccupied aerial vehicles (UAVs), commonly referred to as drones, are increasingly incorporated into the toolboxes of many ecologists [1–4]. These tools provide researchers with an aerial platform to monitor organisms that are challenging to observe directly [5]. For this reason, UAVs are frequently used to study many marine taxa, including sea turtles, crocodiles, sharks, and whales (e.g., [6–9]). Moreover, technological innovation means that novel sensors, such as thermal cameras or LiDAR, are continually incorporated into the available sensor array for UAVs and, in turn, this increases the quantity and type of data these devices can collect [10].

Morphometric data can provide valuable insights into a species’ ecology and population status following disturbance or recovery efforts [11–15]. However, accurately measure body size can be challenging when using UAVs [1–4]. This might initially appear paradoxical as most modern UAVs can measure their altitude, and so if the width of the camera’s field of view at a given altitude is known, then it is possible to determine the size of an object within the viewing window. Indeed, such altimetry-based methods have proven useful for measuring large marine animals, such as whales and elasmobranchs [16–18]. For smaller marine animals (<2m), an error of ∼5 cm can translate into a higher error rate (i.e. greater percent error) and can mean the difference between classifying an individual as an immature or mature, but for larger animals, such as whales, a ∼5 cm error may be negligible. Further, issues associated with image resolution and uncertainty regarding the exact altitude of the drone can lead to higher measurement error [19–21]. Regardless, problems associated with image resolution and uncertainty regarding the exact distance of the camera relative to the animal of interest may ultimately reduce measurement accuracy. This is especially pertinent when studying marine megafauna whose depth in the water column may not be readily measurable or if the animal is non-orthogonal to the camera (i.e. diving or surfacing during video acquisition). To address this issue, some studies have suggested using LiDAR or structure-from-motion (SfM) technology to assess body size [10, 22]. These tools have proven highly practical, but may still produce measurement errors up to 6 cm [23]. Additionally, depending on the choice of altimeter (barometer or laser altimeter), uncertainty related to predicted body length of whales decreased with altitude, suggesting a relationship with error and altitude [19]. There are also GPS-based systems, such as Real-Time Kinematic (RTK) drones, that have on-board GNSS RTK receivers that gather data from satellites and a stationary base (ground) station to more accurately correct image location, in real time as it flies [24]. However, if field surveys are conducted offshore or in remote locations, a portable base station may be required to maximize the accuracy of an RTK drone. RTK drones are all quite expensive, which may be prohibitive for marine wildlife studies with limited budgets. As such, there is an opportunity to explore other methods that may be more suitable to boat-based surveys, such as the use of UAV-mounted stereo-video cameras (SVCs). We provide a comparison of the advantages and disadvantages of using altimetry versus SVC-based methods for morphometric data collection from vessel-based aerial surveys in Table 1 [25]. Notably, aerial SVCs have several advantages for this particular application, such as not depending on altimetry data, nor are animals required to be orthogonal to the cameras.

**Table 1:** Comparison of the altimetry and stereo-video camera methods for morphometric measurements.

Method	Advantages	Disadvantages
*Altimetry-Based*	• Single camera	• May need additional sensors for higher accuracy (e.g., altimeter) which also require calibration (e.g., altimeter drift)
	• Good accuracy from high altitude	• Object must be orthogonal to camera or requires ortho-correction
		• May require base stations (RTK drones)
*SVC-Based*	• Altitude data not needed	• Two or more cameras needed
	• Can calibrate for multiple zoom configurations	• Accuracy affected by object’s distance from camera
	• Can measure objects non-orthogonal to camera (e.g., diving, submerged in water, etc.)	

SVCs consist of two cameras that collect video footage of the same object, but from different, albeit fixed, angles. If the distance between each camera and their relative angle to each other is also known, then accurate object size data can be extracted from resulting images using trigonometry [26]. Hand-held SVCs are frequently used to remotely measure marine animals in-water, including sharks [27], bony fish [28], whales [29], and turtles [30, 31]; however, there are currently no studies that have explored the capabilities of UAV-mounted SVCs to measure body size in marine animals. In fact, some studies have suggested that SVCs are prohibitively large and cumbersome to be used for this purpose [10, 22, 23, 32]. Nevertheless, technical advancements in UAVs, batteries, and advanced manufacturing approaches, such as 3D printing, provide new opportunities for the design of novel, stereo-video equipped UAVs.

### Preliminary stereo-video camera system design considerations

Our primary objective was to design, build, and test a stand-alone SVC fixture that could be mounted to a commercial UAV, and be used for non-contact measurement of underwater targets. Key design considerations and constraints included lightweight design, compatibility with SeaGIS software (i.e., CAL and EventMeasure, SeaGIS Pty Ltd., Bacchus Marsh, Victoria, Australia), and adaptability to commercially-available UAVs. During the design phase of the proof-of-concept prototype, a key challenge was mounting the SVC system in a manner that minimally impacted the UAV’s flight dynamics, and maximized flight time. During the concept generation phase, adding a SVC gimbal mount was considered, however testing revealed that dynamically rotating the SVC may destabilize the UAV, depending on the SVC to UAV size ratio. The current fixed camera design utilizes the UAV’s planar rotation ability, parallel to the horizon, which allows the user to keep moving objects (e.g., sea turtles) within the SVC’s point of view. In future work, we will examine the trade-offs between SVC camera distance, SVC gimbal mounting, and reduction in UAV flight performance. We also tested the impact of filming an underwater object from the air to account for image distortion due to refraction [33]. However, we did not account for the relationship between water salinity and the corresponding index of refraction, as the index of refraction varies very little across freshwater and sea water. For example, at a wavelength of 546.1 nm and 30°C water temperature, the index of refraction is 1.39807 for seawater at 35% salinity and 1.38919 for fresh water at 0% salinity [34, 35]. This is significantly less than the measurement error from single camera UAV systems, and we considered this negligible for our application.

Here, we present a functional proof-of-concept for a SVC equipped UAV. We also test the use of this device to accurately measure body size in marine species. Such a tool has the potential to facilitate data collection for many marine species that lack body size data for certain life stages. For example, a gap analysis focused on sea turtle juvenile life stages found that studies on juvenile leatherback (*Dermochelys coriacea*) and hawksbill (*Eretmochelys imbricata*) sea turtles, in particular, are lacking globally, and that all species in the Indian, South Pacific and South Atlantic Oceans are data poor [36]. However, with sufficient body length data, it is possible to conduct length-based population assessments that can help inform population trends, age-class specific survival rates, size-at-maturity, and other critical vital rates [13, 37–40].

## Materials and methods

### Ethics statement

Research conducted in Eleuthera was permitted by the Bahamian Department of Marine Resources (permit MA&MR/FIS/9). All researchers involved in animal handling obtained IACUC training and were permitted through the University of West Florida. In figures with photographs of researchers participating in testing the SVC-UAV system, the individual who is identifiable has given written informed consent (as outlined in PLOS consent form) to publish.

### Testing the effect of refraction on SVC measurement accuracy

**Fig 1:**
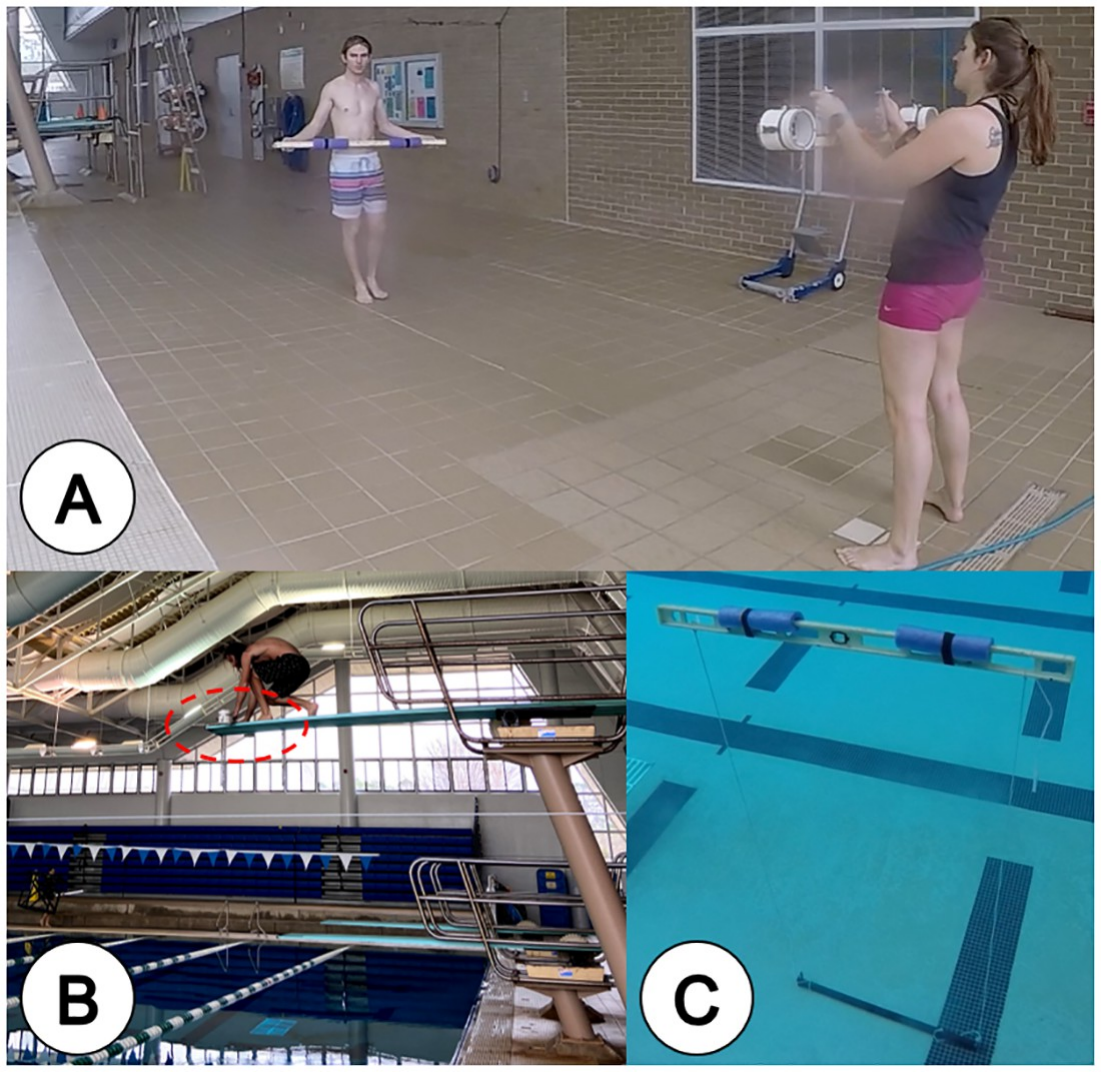
Examining the feasibility of using stereo-video footage collected from the air to measure objects below the water surface. (A) Calibrating the SeaGIS SVC to a custom scale bar before testing. (B) SeaGIS swimmable stereo-video camera on the high dive, stereo-video camera in red dashed outline. (C) Underwater scale bar testing mechanism at 5.0 m depth. Testing was conducted at the University of West Florida Aquatic Center.

To examine how refraction affects SVC measurement accuracy, a SeaGIS swimmable SVC system was fixed to the high dive of a pool to record a fixed length object at increasing depths in the pool (Fig 1). The swimmable system uses two GoPro Hero 5 Black cameras set to 1920 x 1080 video format, medium field of view, and 30 frames/sec. The two cameras were spaced with their lenses 0.8 m apart, and were inwardly converged at 4°. Pool testing was conducted at the University of West Florida Aquatic Center.

Prior to in-water measurements, the SVC was calibrated in the air using the method described by Harvey and Shortis [41]. In short, the calibration used multiple images of a 3D aluminum cuboid frame marked with 56 precisely known reference points. The locations of these points were measured from multiple images taken from 20 standardized orientations of the calibration cube. Images were analyzed in SeaGIS CAL software v.3.23 to calibrate both the internal and external parameters of the SVC [42]. The calibration calculates the camera parameters, including the focal lengths (i.e., the distance that the lens converges light) of the cameras.

We used a custom scale bar with identifying distance markers (Length 1 = 0.4499 m, Length 2 = 0.7653 m, and total bar length = 1.2152 m) as the measurement object. The scale bar was placed in the pool and leveled horizontally with floats. Cords were connected to each side and vertically attached to a steel beam that allowed the bar’s depth to be adjusted by wrapping the cord around the beam. Video was collected from the high-dive (3 m from surface), with the swimmable SVC system, from 0 (surface) to 1.07 m depth at 0.15 m increments. The distance (altitude) between the SVC and the measuring bar ranged from 2.9—5.2 m.

### Unoccupied aerial vehicle platform and stereo-video camera mounting system design

**Fig 2:**
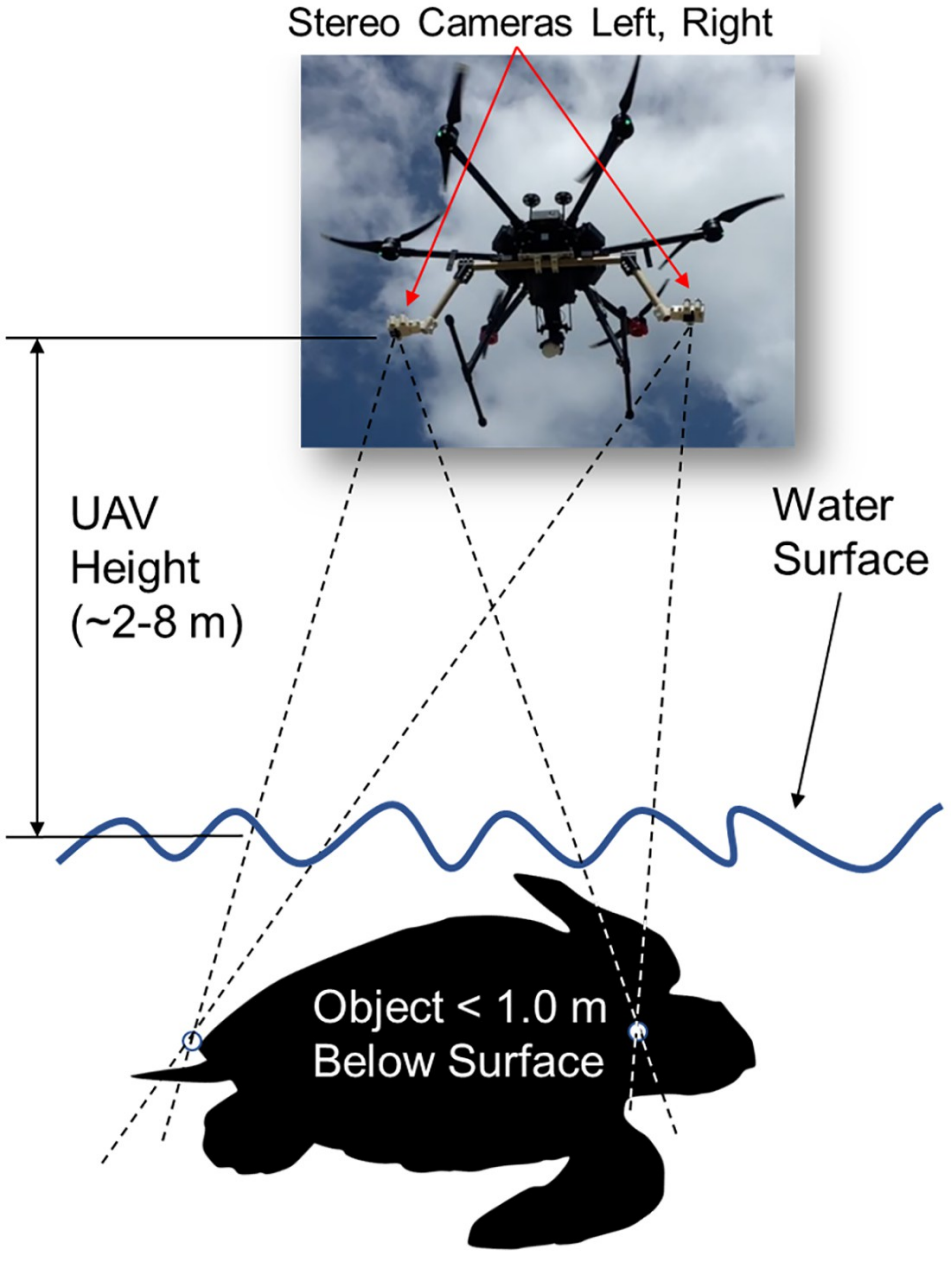
Conceptual overview of stereo-video camera mounting to an unoccupied aerial vehicle. Dashed lines indicate the convergence points of left and right cameras on object, relative to UAV height above the water surface and underwater object. (Turtle silhouette image by Spotila and Chatterji—no copyright).

**Fig 3:**
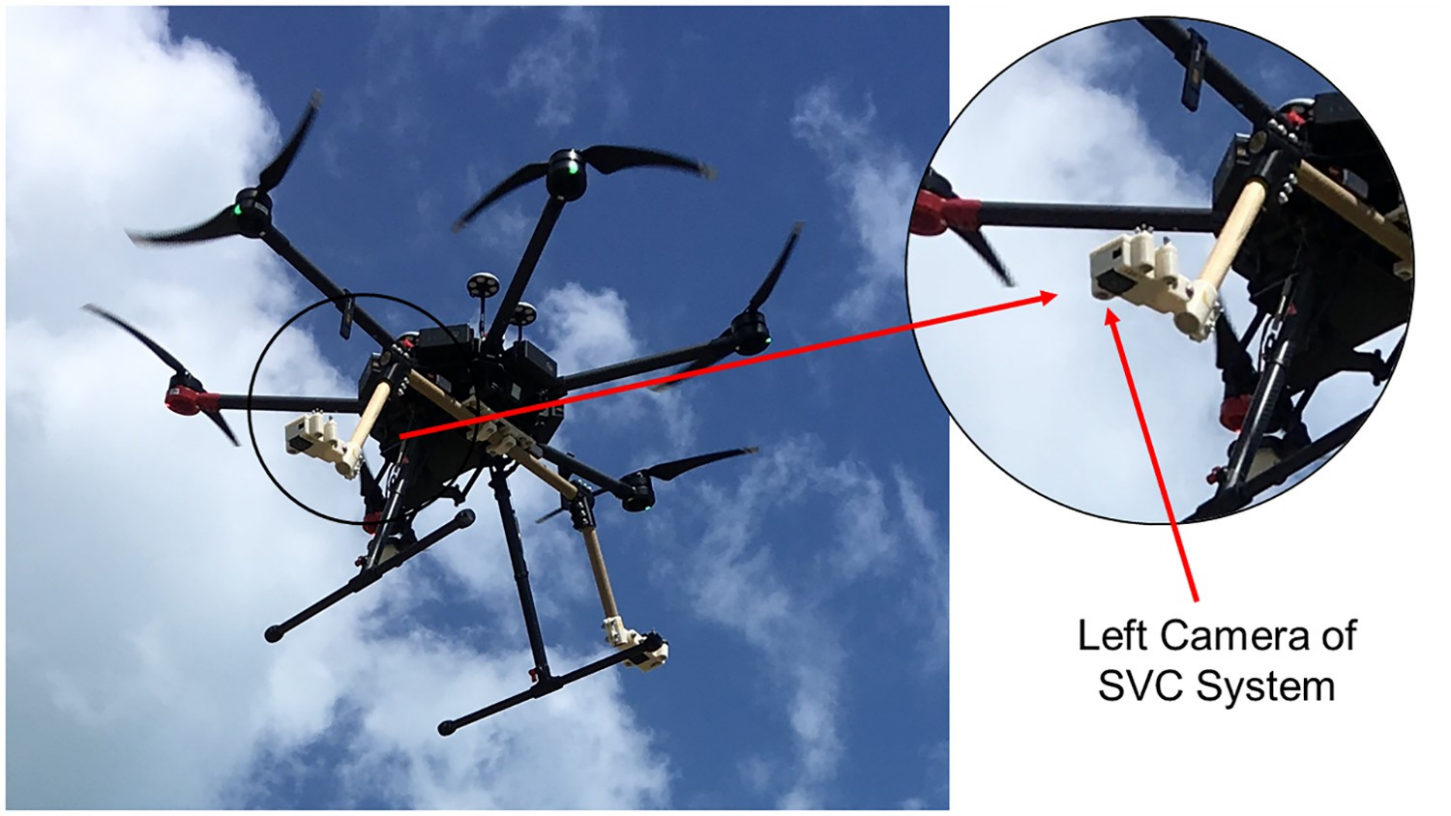
Unoccupied aerial vehicle in flight with stereo-video camera system mounted, and left camera highlighted.

**Fig 4:**
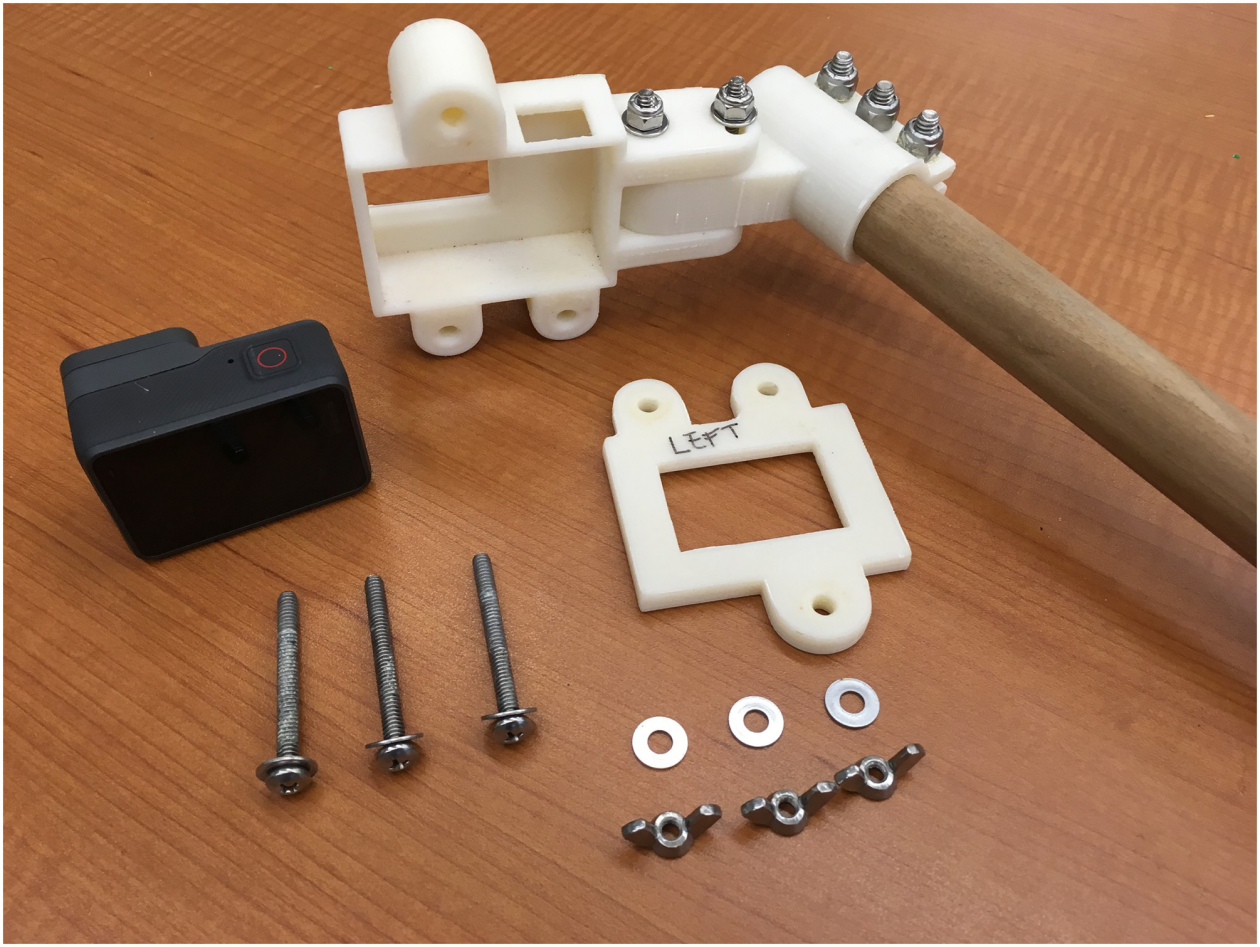
Stereo-video camera housing. Custom ABS plastic 3D printed housing, which allows access to camera functions for the GoPro Hero 5 Black camera. Housing is fastened using stainless steel screws and wingnuts for corrosion resistance.

We selected the DJI Matrice 600 to carry the custom SVC based on its large payload capacity of 15.5 kg, and maximum flight time of 32 min [43]. Figs 2 and 3 provide visual context to the SVC design, mounting, and operation. The SVC system was comprised of a custom built frame and two camera housings with GoPro Hero 5 Black cameras (Fig 4). The frame was made from solid 2.54 cm diameter pine wood dowels. We used pine as it has a high strength-to-weight ratio; however, future work will explore using structural composites, such as carbon fiber [44]. The cameras were mounted to the frame with unique left and right adjustable three piece housings (Fig 4). These were designed in SOLIDWORKS 2018 (Dassault Systemes) before being 3D printed with acrylonitrile butadiene styrene (ABS) plastic using an Airwolf EVO printer and assembled with stainless steel fasteners for corrosion resistance. The camera power buttons and touchscreens could be accessed when in the housings. The camera housings were a fixed distance of ∼0.8 m apart and the convergence of their focal point could be adjusted from ∼0°to ∼15°for calibration [41, 45]. For this design and subsequent experiments, the cameras were inwardly converged at approximately ∼4°. Each camera was set to 1080p video format, medium field of view, and 30 frames/sec, based on the video requirements of the SeaGIS EventMeasure software v.5.22, however higher resolution video will be tested in the future. We followed the same EventMeasure protocol for calibration as the swimmable in-water SVC system with the sole exception that we used calibration settings for measuring in air rather than water.

### Bahamas field trials: Study site, animal capture, and filming techniques

We tested the accuracy of the SVC-UAV on juvenile green sea turtles (*Chelonia mydas*) and nurse sharks (*Ginglymostoma cirratum*) in July 2019 in the waters off Cape Eleuthera, the Bahamas (24°49’54“N, 76°19’45“W). We collected physical measurements by hand and used those as the control to quantify the accuracy of the SVC-UAV system. Sea turtles were captured using a rodeo technique [46]. After hand capture, turtles were hauled on-board the boat to measure the maximum straight carapace length (SCL), from the nuchal scute to the posterior tip of a supracaudal scute (±0.1 cm; SCL); and curved carapace length (CCL). SCL was measured using metal calipers and CCL was measured using a flexible tape measure [46]. After data collection (which usually lasted less than 15 min), turtles were released within 100 m of their initial capture location. In contrast, nurse sharks were captured using a baited hand-line. Once hooked, the sharks were brought alongside the boat and a rope with a noose was looped around the caudal fin. Using the hand-line that was still hooked in the shark’s mouth as well as the rope around the caudal fin, it was possible to keep the shark stable and immobile. During this time, total length (TL; snout to tip of caudal fin) of the shark was measured using a flexible tape measure [47, 48]. The shark was released within 10 min after capture by cutting the hook and releasing the noose around the caudal fin.

To record the free-swimming animals via the SVC-UAV, we first synchronized the cameras using an unique initial cue (such as hand clapping or fingers touching) [41]. The UAV was then positioned directly above the animal at ∼2–8 m altitude. Notably, in practice many studies use a minimum altitude of 30 m to avoid interaction with sensitive avian species [49]. We did not specifically control altitude during the field trial, as our objective was to simulate measuring free swimming animals in the field where scientists may need to opportunistically measure animals under a variety of altitudes. Further, we did not measure altitude directly, but rather used the SVC image analysis software to measure range, which is the distance between the cameras and the object being measured, and we accounted for range in the statistical analysis (see sections Stereo-video image analysis and Statistical Analysis). As our initial proof-of-concept design was tested at relatively low altitudes, our future work will explore cameras with a 4X zoom, which may allow us to use this method at higher altitudes. The animals were released after hand measurements in the general direction of the SVC-UAV or off to the side of the boat to allow for adequate video footage to be obtained (i.e., obtain enough video frames where the nuchal and supracaudal scutes were visible). The animals were followed with SVC-UAV until they were no longer visible, such as the animal dove deeper than visible from the surface or swam outside the viewing area of the single gimbal camera, which we viewed live on a computer tablet that we used to fly the UAV. The SVC was synchronized for a second time upon retrieval, as a back-up.

We simultaneously filmed the released animals in the water using the in-water swimmable stereo-video camera, the same system used in the pool testing. This system has been previously established to remotely measure body lengths with reasonably high accuracy [30, 50]. We conducted this additional measurement simultaneously so as to provide an additional comparison for the accuracy of the SVC-UAV compared to a more traditional in-water remote measurement method.

### Stereo-video image analysis

**Fig 5:**
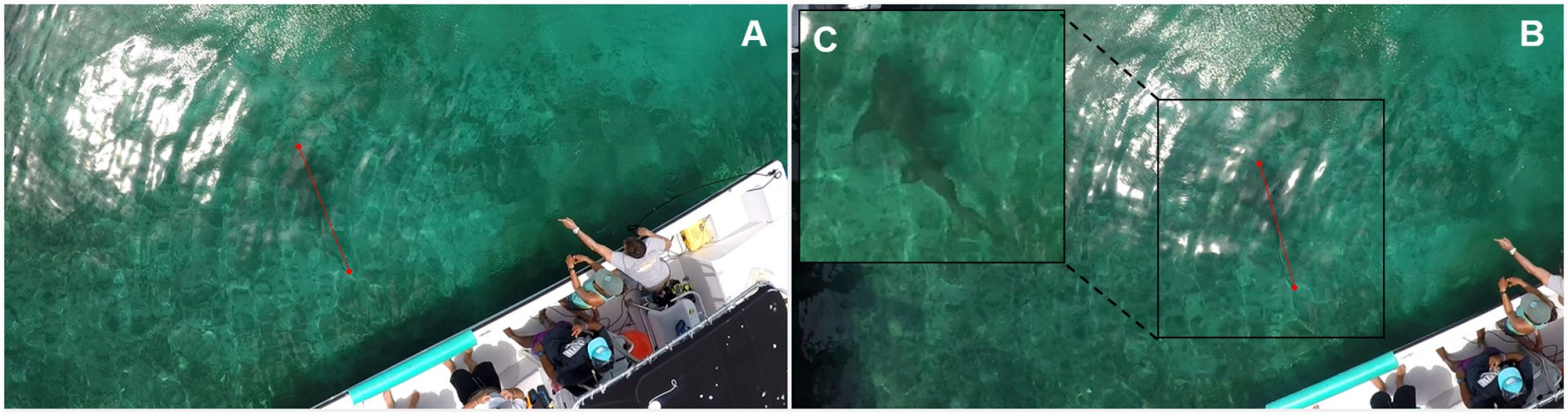
Stereo-video camera software interface. (A) Left and (B) right camera views of a nurse shark (*Ginglymostoma cirratum*) measured for total length, and (C) zoomed-in image of the right camera image of the shark. In (C), the photo has been enhanced for contrast and to sharpen edges to aid in shark visibility. Red lines indicate the measurement vectors for total length.

We analyzed video footage using SeaGIS EventMeasure software v.5.22. EventMeasure produces length measurements (i.e., the distance between two fixed points) along with an estimate of measurement error. The resulting video footage from the two cameras was synchronized using the unique initial cue, and the same two measurement points were selected within an image pair from the right and left cameras (Fig 5) [50]. To maintain acceptable precision, the root mean square (RMS) error should be < 20 mm [41]. Here, the RMS error calculates the exactness of the 3D intersection between the image pairs [41]. To reduce measurement error from individual video frames, the average of five distance measurements were used as the final value, which is standard practice for SVC measurements [51]. We measured body length from the same locations as those used for hand measurement. For the nurse shark, which swims with lateral flexion, we took care to select frames where the animal’s body was as straight as possible to avoid additional measurement error.

### Statistical analysis

For both the preliminary pool testing and field trials, we calculated several different metrics of measurement error. We ascertained the mean absolute error between the known length of the scale bar and the measurement collected from the swimmable SVC system. Mean absolute error (MAE) was calculated as:

$$\begin{array}{c} MAE=\frac{1}{n}\sum_{j=1}^{n}\left|\left(v-v_{est}\right)\right| \end{array}$$ (1)

where MAE is the mean absolute error, *v* is the distance between two reference points, and *v_est_* is the estimated distance calculated in EventMeasure. In addition, we also calculated the percent error as:

$$\begin{array}{c} \delta =100*|(v-v_{est})/v| \end{array}$$ (2)

where *δ* is the percent error. We then calculated mean percent error (MPE) across all measurements. We calculated root mean square error (RMSE) as well as:

$$\begin{array}{c} RMSE=\sqrt{\frac{1}{n}\sum_{j=1}^{n}{\left(v-v_{est}\right)}^{2}} \end{array}$$ (3)

For the pool testing, we also examined the relationship of the MAE of the measurements and depth using a generalized linear model (GLM) with a gamma distribution. We used this distribution as there were some minor departures of the residuals from a normal distribution as determined by examining the quantile-quantile plot and comparing the fitted values to the residuals.

For the field trials, we evaluated accuracy with two different methods. Firstly, we ascertained the absolute error, square error and percent error of measurement by hand (the “true” measurement) and the measurement collected from the SVC-UAV system. We calculated the MAE, RMSE, and MPE to assess the relationship between the SVC system measurements to the “true” measurement achieved with calipers for sea turtles and measuring tape for the nurse shark using Eqs 1–3. Secondly, to evaluate potential bias in the SVC-UAV measurements, we conducted a linear regression to estimate the slope of the relationship between the SVC and hand measurements for the turtles. We did not use the shark for this analysis as we were only able to measure a single individual. If the slope was equal to one, then this would suggest a perfect agreement between the two measurement types, and also suggests that no bias in measurements is present. We collected five measurements from separate video frames from each individual turtle, which enabled us to use the mean across the measurements for each individual as this is typical practice for measurements collected via stereo-video cameras [51]. We found the assumptions of a linear model were upheld based on visual inspection of the qq plots, the fitted values and the residuals, and the frequency distribution of the residuals.

We also evaluated the relationship with MAE and the distance of the cameras to the sea turtle and the body size of the turtles using a linear mixed model with a repeated effect for multiple measurements for each individual, using the individual measurements from different video frames, as the ranges differed by frame. We used the global model:

$$\begin{array}{c} Absolute\;Error=\beta_{0}+\beta_{1}*Range+\beta_{2}*SCL+\epsilon_{i,j} \end{array}$$ (4)

where *β*_0_ is the intercept, each *β*_*i*_ is the variable-specific contribution to the slope, the range is the distance between the SVC and the turtle, SCL is the straight carapace length calculated in EventMeasure, and the residual error is *ϵ*_*i*,*j*_ ≈ N(0, *ϵ*^2^) of turtle *i*. Once again, we ascertained the assumptions of a linear model were upheld, based on the qq plots, plotting the fitted values to the residuals, and the frequency distribution of the residuals. We performed this analysis on individual measurements from different video frames as the absolute error and range varied by video frame.

Lastly, we assessed the background level of error associated with manual measurements to contextualize the error for the SVC-UAV system. To achieve this, we estimated the measurement error from manual measurements of sea turtle SCL from green sea turtles repeatedly caught and measured during a long-term monitoring survey conducted by the Cape Eleuthera Institute. Using this data-set, we calculated the measurement error from turtles that were re-caught within 30 days (to control for potential size differences due to somatic growth). We calculated error using Eqs 1–3.

In all, we used the information theoretic approach for model selection based on Akaike Information Criterion correction for small sample sizes (AICc) [52, 53]. All analyses were performed in R v.3.6.3 (R Development Core Team 2018) and R Studio v.1.3.1073 (R Studio, Inc.).

## Results

### Initial pool testing

**Fig 6:**
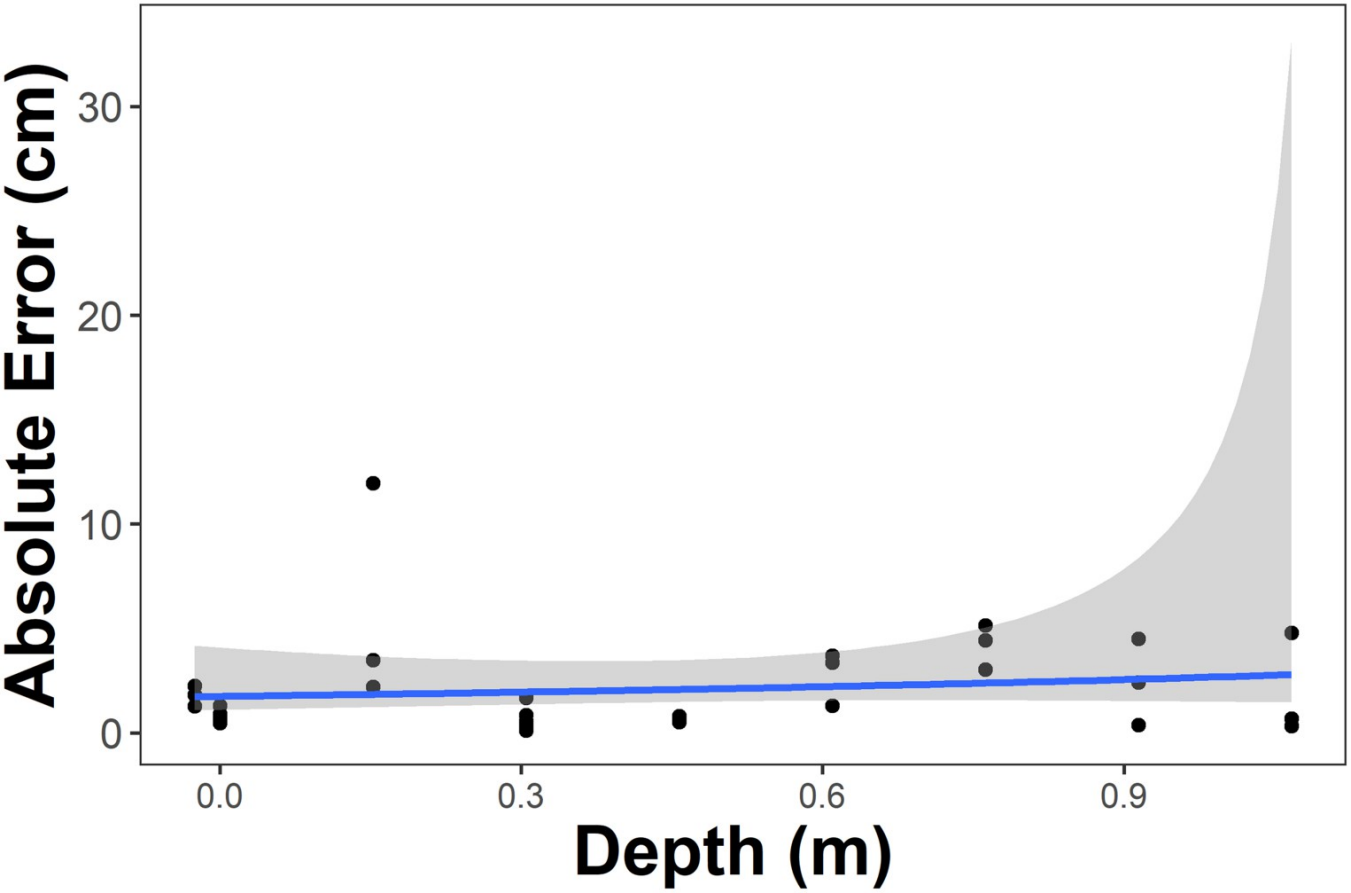
Relationship of absolute error of aerial SVC measurements and depth. The solid line and gray shaded area are the generalized linear regression and standard error, respectively. Depth measurements of <0 indicate measurements made from the camera in the air only, just above the water surface.

In the initial pool experiments, the MAE was 2.12 cm (±0.44 standard error (SE); S1 Table. In comparison, the MAE of measurements made during the scale bar calibration in the air was 0.319 cm (±0.59 SE; S2 Table). We also tested the relationship of the measurement accuracy with depth, and absolute error ranged from 0.1055 cm in 0.3048 m depth to 11.97 cm in 0.1524 m depth, but there was not a linear relationship with depth. Beyond 1.07 m depth, the water refraction was too severe to permit measurement. Based on the GLM (with a gamma distribution) to statistically test the relationship between MAE and depth, the null model (AICc = 118.8) and test model (AICc = 120.2) with depth as the explanatory variable were within 2 *Δ*AICc (S3 Table). This suggests that the null and test models had equivalent fits to the data while balancing model parsimony; thus, depth was not a strong predictor of percent error within the depth range that we collected measurements (Fig 6).

### Field trials

For the Eleuthera field trials, we measured 6 *C. mydas* with body lengths ranging from 27.8—62.2 cm SCL, and 1 *G. cirratum* with a body length of 198 cm TL. The MAE for green sea turtles was 4.44 cm (± 0.99 SE) and for the nurse shark, it was 7.16 cm (no SE available as n = 1, Table 2). Measurement data are provided in the Supporting Information (S3 Table). In comparison, the MAE from the in-water SVC system for green sea turtles was 6.31 cm (±0.980 SE, range 0.391—20.4, n = 6 individuals). In-water measurements were not collected on the shark due to logistical limitations. The MAE for *C. mydas* hand measurements was 3.81 cm (±0.455 SE, range 0–40, n = 96 measurements).

**Table 2:** Measurement accuracy of the stereo-video camera mounted to an unoccupied aerial vehicle and in-water swimmable stereo-video camera.

Species	N	MAE		RMSE		MPE	
		SVC-UAV	SVC	SVC-UAV	SVC	SVC-UAV	SVC
*Chelonia mydas*	6	4.44 ± 0.99	6.31 ± 0.98	4.97 ± 7.67	8.21 ± 18.0	10.59% ± 2.54	1.33% ± 0.16
*Ginglymostoma cirratum*	1	7.16	NA	7.16	NA	3.62%	NA

SVC-UAV = stereo-video camera mounted to an unoccupied aerial vehicle, SVC = in-water swimmable stereo-video camera, MAE = mean absolute error, RMSE = root mean square error, and MPE = mean percent error (± standard error). In-water SVC measurements were not collected for *G. cirratum* and standard errors are not provided for *G. cirratum* as only 1 individual was measured.

**Fig 7:**
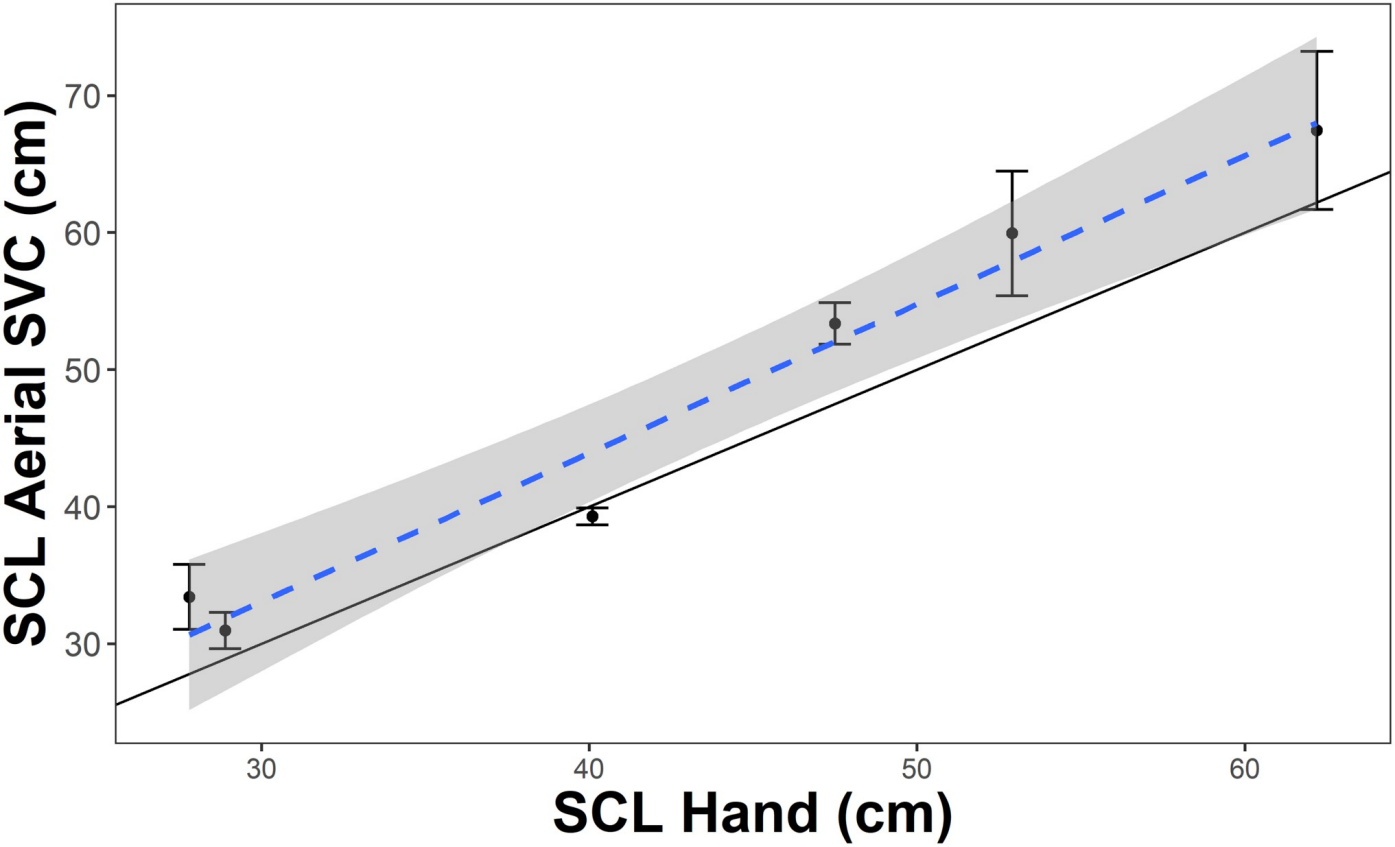
Comparison of hand measurements to SVC-UAV for green sea turtles (n = 6) from the field trial in Eleuthera, Bahamas. The dashed line and gray shaded area are the linear regression and standard error, respectively. The solid black line is the 1:1 line for the hand and SV-UAV measurements; points that fall on this line have equivalent measurements for both methods.

**Fig 8:**
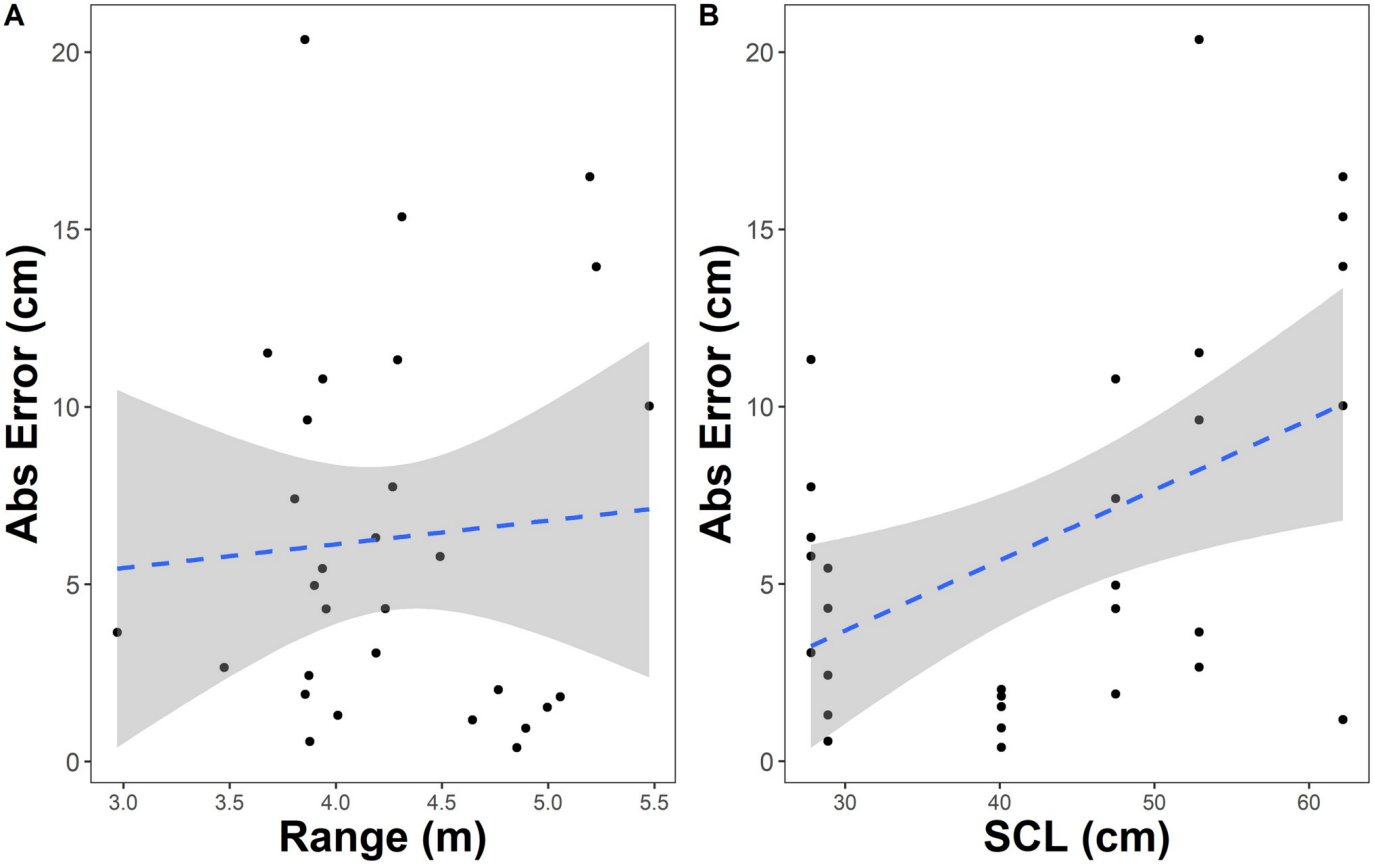
Relationship between absolute error and (A) range and (B) straight carapace length from the stereo-video camera system mounted to an unoccupied aerial vehicle. The dashed line and gray shaded area are the linear regression and standard error, respectively.

We examined the relationship between the SVC-UAV and hand measurements for green sea turtles. The estimated slope was 1.085 (±0.099 SE, 95% confidence interval 0.808—1.36) from a linear model, using the mean of 5 measurements across different frames for each individual. The 95% confidence interval for the slope estimate encompassed 1.0, and this suggests there is not a consistent negative or positive bias to the SVC-UAV measurements for green sea turtles (Fig 7). When we performed model selection on potential predictors of absolute error, range and the sea turtle SCL, the null model was the only model present in the confidence set (S3 Table). This suggests that neither range nor SCL strongly influenced measurement error (Fig 8).

## Discussion

Here, we provide a functional proof-of-concept for mounting an SVC system onto a UAV. The SVC-UAV permits non-invasive measurements of body size in animals. We demonstrate that this method can provide accurate measurements of marine species. However, due to the effects of light refraction, we expect that measurements would only be accurate within the top meter of the water column using our current approach. In addition, since the focal individual must be visible above the water surface, this will exclude measurements of animals that are deeper in the water column. Thus, surface-dwelling or intertidal invertebrates, fish, reptiles, birds and mammals may be excellent candidate species for the current approach, but for now, it may not be practical for demersal and benthic species in deeper water.

Our study underlines the fundamental proof-of-concept of this new technology to remotely measure marine organisms aerially. Our initial pool testing indicated that there was no relationship between measurement error and depth up to the point where water refraction makes the measurement points indiscernible (∼1 m depth). This suggests that as long as an organism is relatively close to the surface, then accurate measurements can be obtained with the SVC-UAV. Moreover, we could not directly measure the depth of the animals in the field trial, because they were being filmed with the SVC-UAV while swimming, so the depth limitations for the field survey may differ from those in the pool testing. However, future work to better understand the correlation between measurement accuracy and depth during field surveys and to address improved measurement with depth would be advantageous. For example, using specialized lenses or underwater image amelioration techniques (e.g., Sea Thru water removal algorithm [54]) may help to improve measurement errors due to refraction, though these would need to be validated directly. Further, Odzer et al. [55] found that in using UAVs to detect marine organisms, depth, seagrass cover, and glare affected model sea turtle detections. Secondly, we found no relationship between the measurement error of SCL measurements and the distance between the SVC-UAV and the sea turtles (Fig 8). It is possible that within the range of our flight altitude (2—8 m), measurement error generally did not increase. This follows with other studies that have investigated the relationship between object distance and measurement accuracy [30, 56]. Also, body size was not a strong predictor of measurement error, and this also suggests the SVC-UAV system is not limited in the size of organisms that can be measured, and would be useful across a range of animal sizes. However, as there was a fair amount of variance in the measurement errors for each turtle and relatively low sample size, the model selection process may not have been able to balance model fit to the data with potentially informative predictors of measurement error. Hopefully, this will be resolved in future iterations of the SVC-UAV and additional validation studies with larger sample sizes. In practice, the use of the mean of multiple measurements across different video frames of the same individual reduces measurement error; indeed, some practitioners recommend using the mean of 10 measurements from unique video frames [51]. We used a mean of 5 measurements here, because some of our video files were damaged, most likely due to the heat and intense sunlight during the field study in Eleuthera in July. It would serve future researchers well to shade the cameras as much as practicable.

Our results indicated that the MAE for the UAV-SVC system for measuring both sea turtles and sharks was 4.44 cm and 7.16 cm, respectively. Notably, this error was similar to the error from physical hand measurements (MAE = 3.81 cm) of juvenile sea turtles in the Bahamas. In general, there are several reasons behind this degree of error, including picture quality, the effect of surface ripples in distorting the body shape, and light refraction [55]. However, all types of measurement, including physically measuring objects, have some degree of error. So, it is important to contextualize this error as it compares to error associated with more conventional measurements, such as direct hand measurements or in-water SVC systems. In this study, the MAE for the handheld SVC was 6.31 cm (percent error 1.34% ± 0.16 SE) and Siegfried et al. [30] measured percent error to be 0.98% (±0.01). In addition, Hodgson et al. [57] reported the estimated slope for a linear regression comparing standard length from physical and photogrammetric measurements was 1.02 (95% CI 0.98—1.07) when measuring Australian sea lions (*Neophoca cinerea*), which is similar to our slope estimate for green sea turtles of 1.0855 (±0.099 SE). Dawson et al. [23] tested the accuracy of photogrammetry using a UAV equipped with a gimballed micro4/3 camera and 25 mm lens with the objective of measuring southern right whales (*Eubalaena australis*). The authors used a floating reference target, flight altitudes ranged 16—30 m, and the UAV single gimbal camera had a mean percent error of 1.0% (SD = 4.9 cm). Our SVC-UAV system has slightly higher errors than other commercial systems, however, additional options for photogrammetry allow scientists to select the best system for their study objectives and budgetary limitations (i.e., Table 1).

While our measurement errors are slightly higher than other remote UAV methods (e.g., [23, 57]), our approach provides a baseline to assess the feasibility of SVC-UAV systems, and will aid future improvements to SVC-equipped UAVs. When accuracy is of vital importance, then direct measurements and hand-held SVC systems will provide the most accurate data. Our SVC-UAV system is advantageous over single-camera UAVs, because it is low cost, and does not require animals to be at the surface nor orthogonal to the cameras. Yet, the UAV-SVC would also be a good option when traditional sampling or in-water sampling is not feasible due to logistical or safety constraints or larger sample sizes are required than can be achieved by hand-capture. For example, where water bodies are too shallow to access by boat, or in-water operations are not safe due to tidal currents, fishing activities, etc. If populations are remote or research programs have limited field crew capacity, then the aerial SVC may also be a good solution, especially as drones can survey large areas quickly [32]. In addition, aerial SVCs will also be practical if a lower level of accuracy is acceptable. For example, for some data-poor populations, simply being able to assign individuals to size categories (e.g., 5 cm size bins) or life stages (e.g., small juvenile, large juvenile, etc.) would be an advancement and so measurements with slightly higher error may be acceptable (e.g. [58]). Further, a sufficiently large enough sample size, given unbiased measurements, would also reduce the need for highly accurate measurements.

In general, MAE was greater in the field test than in the pool test. There are several factors that probably lead to this. First, we measured live animals who were actively swimming, which could induce additional error (i.e., availability of video frames where measurement points were visible). Although, because SCL in turtles is measured across the hard carapace, it seems improbable that body flexion would be an issue, unlike in sharks [27, 30]. Second, the pool testing was performed indoors, where wind, waves and sunlight did not affect the video quality. Furthermore, our analysis of the slope of the SVC-UAV to the manual SCL measurements indicated a possible slight positive bias (although the 95% CI encompassed 1.0). If a slight positive bias was persistent, then it may be possible to apply a correction factor to the SVC-UAV measurements to improve accuracy. However, this should be further tested on a larger data set and in a variety of field conditions. Third, the UAV was actively flying over the turtles and sharks and this produced additional ripples on the water surface that probably increased image distortion. This suggests that there may be an optimal distance between the surface of the water and the maximum altitude of the UAV, although we did not investigate this directly.

The ethical considerations in terms of animal welfare and flight altitude are important to consider as well. For this proof-of-concept study, we flew between 2—8 m altitude as the animals were released from the boat after hand measurement and other data were collected (in support of long-term sea turtle monitoring by the Cape Eleuthera Institute). Some nations currently have altitude restrictions for UAVs studying marine wildlife. For example, in the United States, the National Oceanographic and Atmospheric Agency (NOAA) requires the minimum altitude for UAV flights for marine mammals for public wildlife viewing to be 304.8 m (although a lower minimum altitude could be permitted as part of a scientific research permit [59]). In general, more research needs to be conducted to understand responses of marine wildlife to UAVs. Bevan *et al.* [9] studied responses of marine wildlife to UAVs, and they encountered sea turtles in nearshore, reef and nesting beach habitats, and had flights that ranged from 5—30 m altitude. They observed one hawksbill sea turtle (*Eretmocheys imbricata*) that responded to the UAV by increasing flipper strokes, as the UAV flew at 9 m altitude, while other turtles with UAVs flying at lower altitudes displayed no behavioral responses. Anecdotally, we have noticed that if sea turtles are swimming below the surface, they do not display behavioral responses to UAVs, regardless of the UAV altitude, but they have mixed responses when they surface to breathe and can hear the UAV. In addition, while there have been limited studies to assess the impacts of UAV noise on sharks, it appears that they are unlikely to be negatively impacted based on how noise travels through water [49, 60]. Future work to identify the ideal altitude to fly the UAV to balance the trade-offs in measurement accuracy, minimize behavioral responses, and reduce surface water disturbance would help to resolve this issue.

Based on both the results and lessons-learned from our current SVC-UAV design, we plan to explore several concurrent paths for future work. First, the existing SVC system weighs 1.4 kg, which limits commercial UAV adaptability due to payload capacity and flight time constraints. New SVC designs will consider reducing weight with composite tubing, carbon fiber embedded 3D printing, and polymer fasteners. Second, the focal length of the GoPro cameras, and lack of a zoom function require the UAV to fly relatively close to the animal (e.g., 2–8 m altitude). Exploring alternative camera options could allow filming from greater altitudes, and minimize disturbance to the animals and the water surface. Finally, the existing camera calibration process using the rigid cube is not practical when traveling to remote field locations, especially if the UAV experiences any mechanical damage during field deployment, which would then require re-calibration. Creating a more portable and automated camera calibration process could greatly improve the SVC-UAV’s usability.

## Supporting information

S1 Table Pool testing stereo-video camera—UAV by depth data. (CSV)

S2 Table Air testing stereo-video camera—UAV data. (CSV)

S3 Table Model selection tables for pool testing—Absolute error by depth GLM and field testing absolute error and SCL and depth GLMM. (CSV)

S4 Table Body length stereo-video camera—UAV data. (XLSX)
